# Case report: Ventricular primary central nervous system lymphoma with partial hypointensity on diffusion-weighted imaging

**DOI:** 10.3389/fneur.2022.923206

**Published:** 2022-10-21

**Authors:** Xintong Li, Hua Xiong

**Affiliations:** ^1^Department of Radiology, Chongqing General Hospital, Chongqing, China; ^2^Department of Radiology, People's Hospital of Shapingba District, Chongqing, China

**Keywords:** primary central nervous system lymphoma, third ventricle, diffusion weighted imaging, T2 blackout effect, case report, ventricular PCNSL

## Abstract

**Introduction:**

Primary central nervous system lymphoma (PCNSL) is infrequent and represents 3. 1% of primary brain tumors. And the lesions that are restricted to the ventricular system, particularly the third ventricle, are even rarer. There are few pieces of literature or case reports to date. We report a case of PCNSL with partial hypointense on diffusion-weighted imaging (DWI) located in the lateral and third ventricles. Then we reviewed almost all case reports of ventricular PCNSLs in the last 20 years, discuss the imaging presentation, other ventricular tumors with similar imaging findings, and primary treatment measures.

**Case presentation:**

A 78-year-old man presented with memory loss and poor responsiveness for one week without obvious precipitating factors. Magnetic resonance imaging (MRI) showed lesions in the third ventricle and left lateral ventricles, which were slightly hypointense on T1-weighted imaging (T1WI), and isointense to slightly hypointense on T2-weighted imaging (T2WI). On DWI, the left lateral ventricular lesion was hyperintense, while the third ventricular lesion was hypointense. After the surgical procedure, the pathology and immunohistochemistry revealed diffuse large B-cell lymphoma (DLBCL).

**Conclusions:**

Ventricular PCNSL is quite rare, and may be confused with other tumors in the same position. However, PCNSL differs from other central nervous system tumors in that it is primarily treated with chemotherapy and/or radiation therapy. So, it is important to recognize PCNSL and differentiate it from other tumors, considering its implications for management planning.

## Introduction

Primary central nervous system lymphoma (PCNSL) is an extra-nodal variant of non-Hodgkin lymphoma with disease limited to the brain, spinal cord, leptomeninges, or eyes, without evidence of systemic involvement (1). It represents 3.1% of primary brain tumors. Most PCNSLs are diffuse large B-cell lymphomas with different biological behavior, management, and prognosis compared with systemic diffuse large B-cell lymphomas (DLBCL) (2). PCNSLs differ between immunocompromised (those with acquired immunodeficiency syndromes) and immunocompetent patients. In the immunocompetent population with PCNSL, lesions are usually solitary and located in the brain parenchyma (3).

The vast majority of PCNSLs are supratentorial, but they may arise from other areas of the central nervous system (CNS), including the brainstem, cerebellum, leptomeninges, and rarely the spinal cord or structures of the eye. It is extremely rare that the lesions are confined to the ventricular system, especially the third ventricle. To date, the related clinical and imaging findings regarding the ventricular PCNSL are confined to single case reports and short series (Supplementary Table 1) (1, 4–13). Here, we report a case of PCNSL located in the third ventricle, the body, and the posterior horn of the lateral ventricle.

## Case presentation

A 78-year-old man was hospitalized to our hospital for a week owing to memory loss and poor responsiveness without obvious precipitating factors. Since the onset, he was in poor appetite and spirit, and occasionally unstable gait. The patient denied headache, dizziness, nausea, or vomiting, but he responded with intermittent headaches in the hospital. He had no ataxia, dystonia, speech disorder, and abnormal immunocompetence. There was also no history of cognitive decline, personality changes, seizures, radiation exposure, systemic infection, or other autoimmune diseases. His family history was unremarkable. Neurological examination did not show focal neurological signs. The patient was diagnosed with prostate cancer 3 years ago and got symptomatic treatment. On further evaluation, a routine blood investigation including HIV was done which was nonreactive.

**Figure 1:**
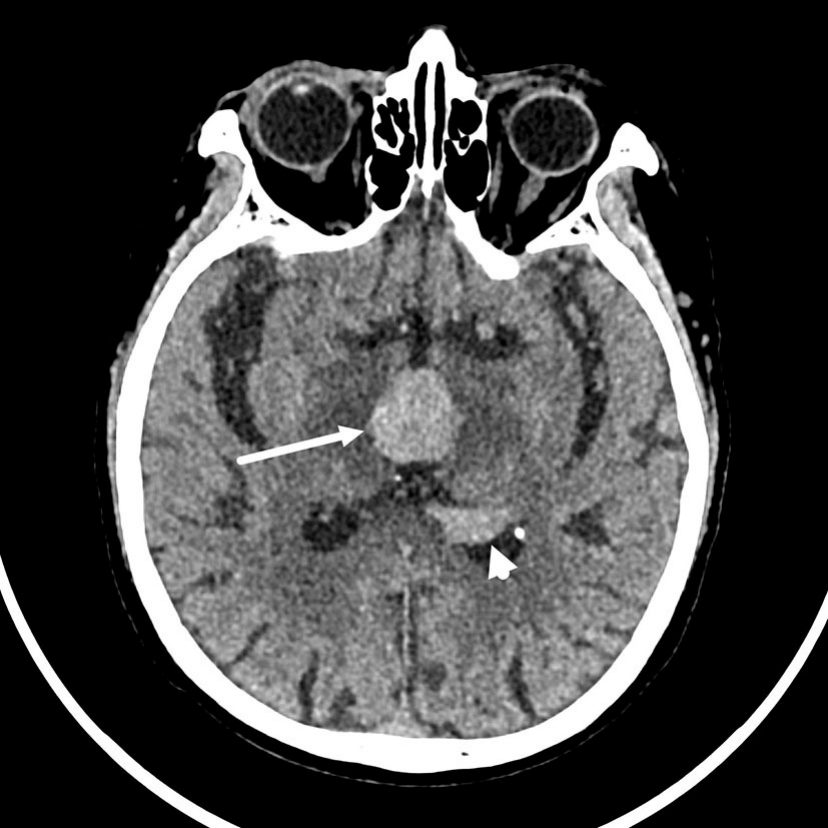
Non-contrast computed tomography (CT) shows that the masses with slight hyper-attenuation were in the third ventricle (the tumor size: 22 mm × 22 mm × 15 mm, CT value: 47HU) (arrow), in the body (not shown) and posterior horn (arrowhead) of the left lateral ventricle.

**Figure 2:**
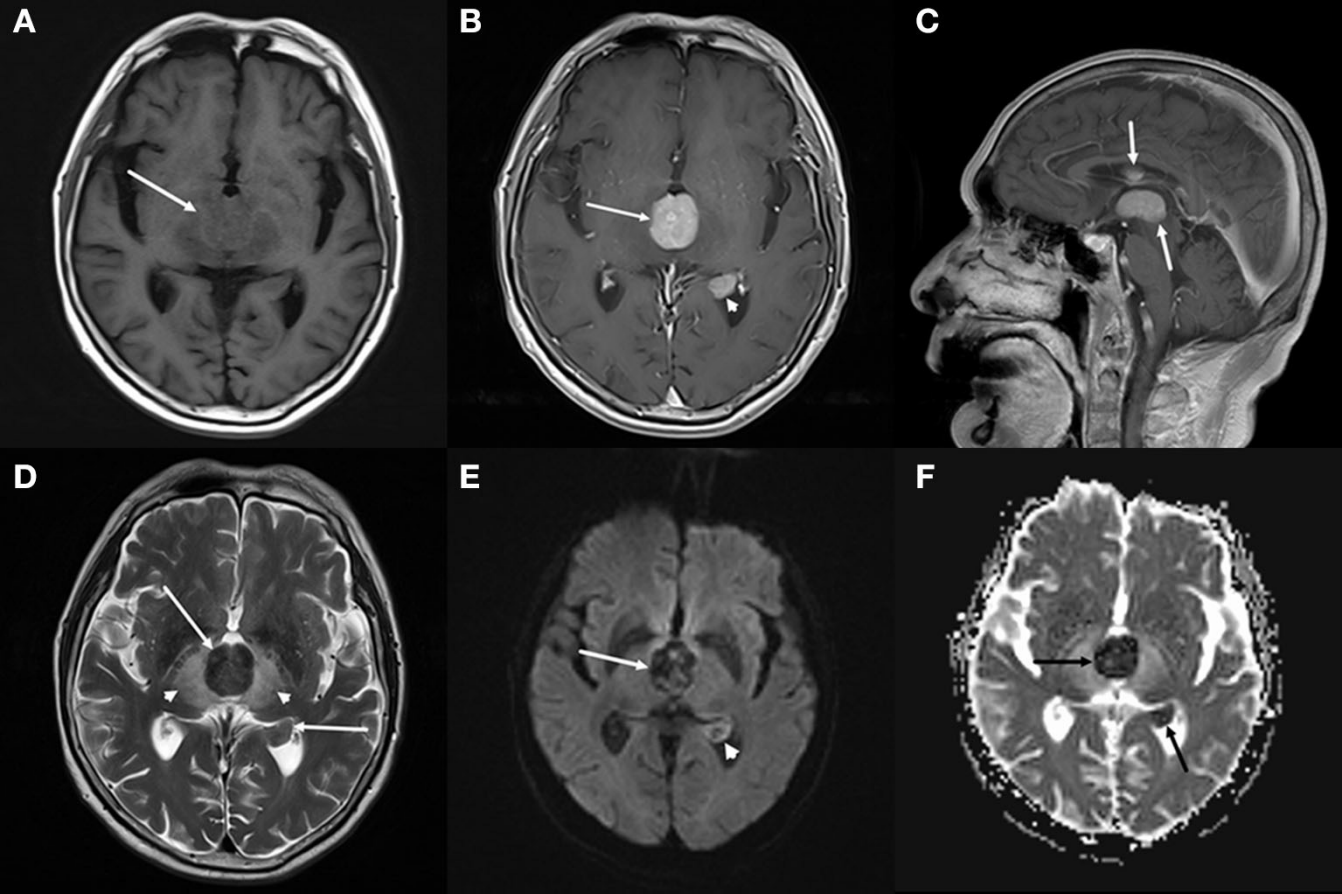
Brain magnetic resonance imaging (MRI). **(A–C)** Axial T1-weighted imaging (T1WI) **(A)** shows that the masses with slight hypointensity are in the third ventricle (arrow) and the posterior horn of the left lateral ventricle. Both of them are significantly enhanced on axial **(B)** and sagittal **(C)** T1WI with contrast. **(D–F)** T2-weighted imaging (T2WI) **(D)** shows the isointense to slightly hypointense masses (arrows), the swollen and hyperintense parenchyma around the third ventricle (arrowheads). Diffusion-weighted imaging (DWI) **(E)** shows a hypointense mass in the third ventricle (arrow) and a slightly hyperintense mass in the lateral ventricle (arrowhead), both of them with low apparent diffusion coefficient (ADC) value (ADC value: 0.425 × 10^3^*mm*^2^/s, 0.628 × 10^3^*mm*^2^/s) (**F**, arrow), all suggesting diffusion restricted.

The brain computed tomography (CT) disclosed three solid masses with slight hyper-attenuation, non-calcification, and non-cystic components. The biggest one was located in the third ventricle and the others were located in the body and posterior horn of the left lateral ventricle (Figure 1), MRI showed multifocal solid lesions in the same regions. They were slightly hypointense on T1WI and isointense to slightly hypointense on T2WI and T2WI dark-fluid images. While on diffusion-weighted imaging (DWI), the third ventricular lesion was hypointense, and the lateral ventricular lesion was slightly hyperintense, both of them with low apparent diffusion coefficient (ADC) values, all suggesting diffusion restricted. Post-enhanced, the ventricular lesions were significantly enhanced. Furthermore, parenchyma around the third ventricle displayed swelling and hyperintense on T2WI. It did not cause ventricular expansion and hydrocephalus above the lesions (Figures 2A–F). He was diagnosed with intraventricular malignancy and based on his history of prostate cancer, the possibility of metastasis was considered clinically.

**Figure 3:**
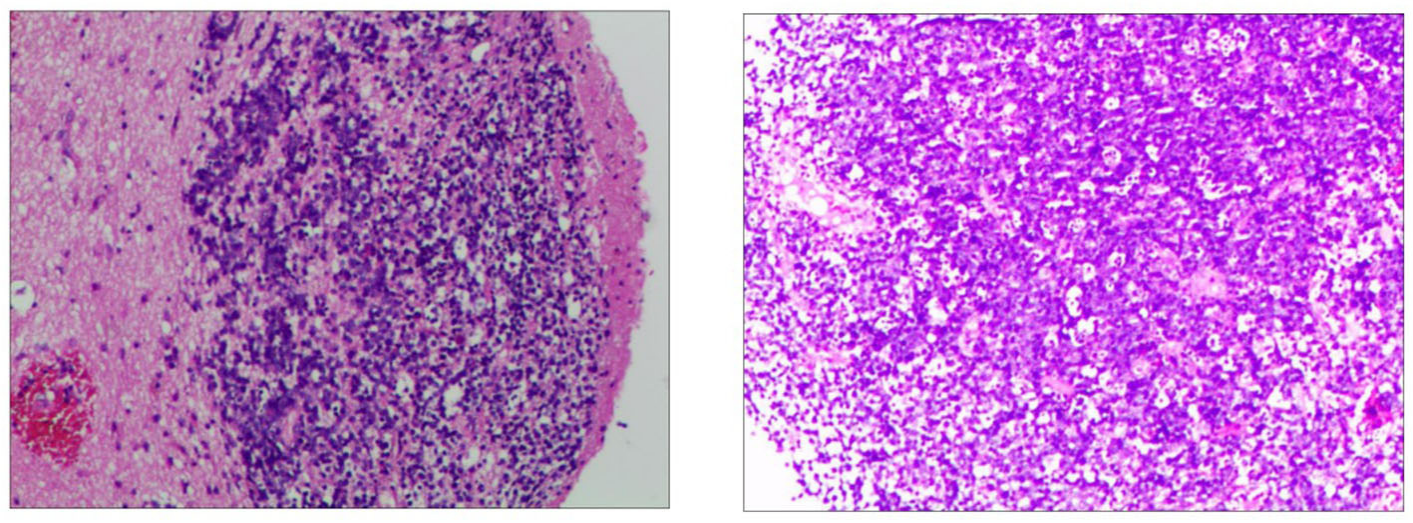
Immunohistochemistry of the third ventricular lesion reveals: CD10 (+), CD20 (+), MUM-1 (+), Bcl-2 (+, 90%), Bcl-6 (+), C-myc (+, 10%), Ki-67 (+, 80%), ALK (–), CD3 (–), CD30 (–), cyclin D1 (–), EBER (–), and P53 (wild-type expression).

In order to determine the nature of the lesion and ensure smooth cerebrospinal fluid circulation in the third ventricle, the patient underwent a subtotal resection of the third ventricle tumor by transcallosal approach. Analysis of a frozen section was consistent with uncertain PCNSL. Immunohistochemistry revealed the tumor cells were positive for CD10, CD20, Bcl-6, MUM-1, C-MYC(10%), Bcl-2(90%), and Ki-67(80%), and negative for CD3, CD30, cyclin D1, ALK, and EBER in situ hybridization (Figure 3). The P53 was wild-type. Thus, the final diagnosis of the third ventricular tumor was DLBCL with a germinal center subtype. Postoperatively, the patient was in a shallow coma, and in poor overall condition. On day 17, he became critically unwell due to respiratory and circulatory failure and arrhythmia. But the families gave up continued resuscitation and requested to be discharged.

## Discussion

Primary central nervous system lymphoma (PCNSL) commonly presents within the brain parenchyma in superficial (subpial) or deep-seated (subependymal location) (13). Most PCNSL involving ventricles are mass lesions originating from the ventricular borders protruding into the internal CSF spaces (12). PCNSL with a pure intraventricular location is rare. Depending on the site of involvement, the patient may present with a large variety of generalized symptoms, such as headache, vomiting, lethargy, memory deterioration, and confusion, as well as lateralizing symptoms such as hemiparesis (6, 14). We reviewed almost all case reports of ventricular PCNSLs in the last 20 years and summarized information on MRI signal, location, and pathological subtype of the cases (Supplementary Table 1). These cases include 18 DLBCLs, 4 high-grade B-cell lymphomas, 4 Burkitt's lymphoma, 2 small lymphocytic lymphomas, and 3 no subtypes provided. DLBCLs constitute the majority of them.

In a series of these ventricular PCNSLs involving various locations, most were T1WI hypointense or isointense, T2WI hypointense or hyperintense, almost all were enhancing. The imaging manifestations are consistent with those of brain parenchyma PCNSL. Except for one case (1) (small lymphocytic lymphoma), all the others (including the current case) were suggestive of restricted diffusion, since the majority of PCNSLs with highly cellular nature. In all cases, only we reported the DWI hypointense of the third ventricle lesion, although the DWI signal intensity reported in part of the cases was not mentioned. It indicates that the DWI signal of this case is particular. We consider that the T2 blackout (TBO) effect may be used to explain the hypointense both on DWI and ADC. TBO, as the opposite of T2 shines through, is a less described effect that reflects the T2 shortening due to paramagnetic substances on DWI. Because DWI primarily is a T2-weighted sequence, T2 changes in tissue may have an influence on DWI (15–17). Exactly, our lesion in the third ventricle is extremely low intensity on T2WI. We speculated that the explanation for T2 hypointense was that the tumor tissue cells had a higher nucleo-plasma ratio than other PCNSLs.

The imaging findings may be often confused with other more common lesions with the same localization when PCNSL is located in the ventricle. A colloid cyst is the most common benign tumor of the third ventricle and accounts for 0.5–1.5% of all primary brain tumors (18). But the colloid cyst on MRI is generally seen as a heterogeneous lesion on T1WI and T2WI. Haddad et al. (7) reported a case of the third ventricular PCNSL mimicking a colloid cyst. Central neurocytoma occurs mainly in young adults. It is situated in the ventricle frequently and may lead to hydrocephalus (19). The tumor usually has a heterogeneous cystic-solid appearance with possible calcification, and always shows unrestricted diffusion in MRI. Meningioma of the ventricle is extremely uncommon and is most commonly found in the lateral ventricles. Radiological features overlap with the common signs of ordinary meningioma. Meningioma is hyperdense with or without calcification on CT, iso-hypointense on T1WI and T2WI, and avid postcontrast enhancement (9, 20). Occasionally, meningioma restricts diffusion, but it is rarely multifocal. Chordoid glioma is a rare central nervous system neoplasm, typically arising from the anterior wall or roof of the third ventricle (21). It is a round or ovoid mass with isointense on T1WI, slightly hyperintense on T2WI, and homogeneous enhancement. Cystic changes and necrosis may be present, but calcification is uncommon (22). As to ventricular ependymoma, 58% of them originate in the fourth ventricle, and 42% in the lateral or third ventricle. Ependymoma is usually iso- to hyper-attenuation, partially calcified mass on non-enhanced CT. On MRI, restricted diffusion is uncommon, the mass appears isointense on T1WI and iso-hyperintense on T2WI, which is a typical heterogeneous signal due to calcification, hemorrhage, and cystic components (23, 24).

There are several features seen on multimodality imaging that help to limit the differential diagnosis to ventricular PCNSLs (10), including multifocality, CT hyper-attenuation, MRI avid postcontrast enhancement, and restricted diffusion. Ball et al. (10) hypothesized that ventricular PCNSL may represent an atypical manifestation of PCNSL that is important to recognize, similar to MALT lymphomas of the dura, lymphomatosis cerebri, and intravascular large B-cell lymphoma (IVLBCL). Determination of whether these represent a pathologically distinct subtype is limited by the small number of reported cases. Thus, our report is significant because it demonstrates and complements another possible DWI signal of ventricular PCNSL.

For PCNSL, the most important role of imaging is directing clinicians to perform a stereotactic biopsy or obtain CSF for a histologic diagnosis and avoid futile attempts at resection (9, 25). In the case reports that mentioned treatment, some patients received gross total or subtotal resection before or after diagnosis, and most of them received radiotherapy or chemotherapy with different regimens after diagnosis. Researches show that radical surgical excision of PCNSL is not warranted, and even partial tumor resection appears to be a negative prognostic factor (2, 14, 26). But another study (2) has suggested surgical intervention may be required when PCNSL leads to increased intracranial pressure, impending herniation, or other neurosurgical emergencies. Our case with subtotal resection is to relieve the intracranial pressure and keep the cerebrospinal fluid circulating well, although the patient's postoperative condition was unsatisfactory in the end. PCNSL is sensitive to both chemotherapy and radiotherapy, and it is better to combine two kinds of treatments according to some studies (2, 9). When PCNSL is suspected, biopsy and chemotherapy/radiotherapy should be mostly suggested in the diagnosis and treatment process (11).

## Conclusion

The ventricular PCNSL is extremely rare and has a similar imaging presentation to other ventricular lesions. Recognition of these unique presentations of PCNSL may help to distinguish such cases from other intraventricular tumors. And it is important for the radiologist to consider PCNSL in the differential diagnosis because the primary treatment is different.

## Data availability statement

The original contributions presented in the study are included in the article/Supplementary material, further inquiries can be directed to the corresponding author/s.

## Ethics statement

The studies involving human participants were reviewed and approved by Chongqing General Hospital. The patients/participants provided their written informed consent to participate in this study. Written informed consent was obtained from the individual(s) for the publication of any potentially identifiable images or data included in this article.

## Author contributions

XL collect the data, investigated the results, and drafted the manuscript. HX reviewed the manuscript for content and ensured the accuracy of medical content. Both authors contributed to the article and approved the submitted version.

## Funding

This research was funded by the Medical Research Program of the Chongqing National Health Commission and Chongqing Science and Technology Bureau, China (grant number 2021MSXM155).

## Conflict of interest

The authors declare that the research was conducted in the absence of any commercial or financial relationships that could be construed as a potential conflict of interest.

## Publisher's note

All claims expressed in this article are solely those of the authors and do not necessarily represent those of their affiliated organizations, or those of the publisher, the editors and the reviewers. Any product that may be evaluated in this article, or claim that may be made by its manufacturer, is not guaranteed or endorsed by the publisher.
