# Supplementary material for: Case report: Ventricular primary central nervous system lymphoma with partial hypointensity on diffusion-weighted imaging
Source: Front Neurol. 2022 Oct 21;13:923206. doi: 10.3389/fneur.2022.923206 (PMC9633983; doi:10.3389/fneur.2022.923206)
Supplement: Supplementary file 1 [file Data_Sheet_1.PDF]

Supplementary Material

Table 1: summary of findings in individuals with ventricular central nervous system lymphoma, including age, sex, MR signal, location and lymphoma subtype.

Author, year	Age/sex	MRI signal	Location	Lymphoma subtype
Haegelen et al., 2001(1)	33/F	T1: isointense	The fourth ventricle	High-grade B-cell lymphoma
Hill et al., 2009(2)	69/M	T1-enhanced: homogenous enhancement	The fourth ventricle	High-grade B-cell lymphoma
Gu et al., 2010(3)	75/F	T1, T2: slight hypointense	The third and the left ventricles	Burkitt's lymphoma
Takasu et al., 2010(4)	71/M	T1: iso-hypointense<br>T2: slight hyperintense	Hypothalamic region and the third ventricle	Burkitt's lymphoma
Sasani et al., 2011(5)	38/M	T1: hypointense<br>T2: hyperintense	Right lateral ventricle	PCNSL
Brar et al., 2012(6)	65/F	T1-enhanced: homogenous enhancement	Occipital horn and trigone of the right lateral ventricle, fourth ventricle	High-grade B-cell lymphoma
Bokhari et al., 2013(7)	50/M	T1-enhanced: homogenous enhancement	The fourth ventricle	High-grade B-cell lymphoma
Rao et al., 2013(8)	59/M	T1: isointense<br>T2: hypointense	The fourth ventricle	PCNSL
Liao et al., 2014(9)	77/M	T2: isointense<br>DWI: slightly hyperintense and slight diffusion restriction	The fourth ventricle	DLBCL
Funaro et al., 2014(10)	68/M	T1, T2: isointense<br>DWI and ADC map: slight diffusion restriction	Whole ventricle	DLBCL
Fabiano et al., 2014(11)	60/F	T1-enhanced: homogenous enhancement	The fourth ventricle	DLBCL
Alabdulsalam et al., 2014(12)	18/M	T1-enhanced: intense enhancement	The fourth ventricle	Burkitt's lymphoma
Grossman et al., 2014(13)	66/M	T1, T2: hypointense<br>T1-enhanced: homogenous enhancement<br>DWI: mild restriction	The fourth ventricle	PCNSL
Zhu et al., 2015(14)	66/M	T1: hypointense<br>T2 FLAIR: hyperintense	The right lateral ventricle and third ventricle	DLBCL
Hsu et al., 2015(15)	61/M	T1-enhanced: homogenous enhancement	The fourth ventricle	DLBCL
Cellina et al., 2015(16)	65/M	T1, T2: hypointense<br>DWI and ADC map: restricted diffusion	The fourth ventricle and hypothalamus	DLBCL
Suri et al., 2015(17)	15/M	T2 FLAIR: hyperintense	The bilateral lateral	DLBCL

		T1-enhanced: homogenous enhancement	ventricle and fourth ventricle	
Liu et al., 2016(18)	6/M	Both T1 and T2: slight hypointense<br>DWI: hyperintense<br>ADC map: hypointense	The fourth ventricle	Burkitt's lymphoma
Haddad et al., 2019(19)	72/F	T1WI, T2WI and FLAIR: hypointense<br>DWI: slight diffusion restriction	The third ventricle	DLBCL
Philippart et al., 2019(20)	71/M	T1-enhanced: homogenous enhancement	Whole ventricular system	DLBCL
Brozovich et al., 2019(21)	65/M	T1-enhanced: homogenous enhancement	The fourth ventricle	DLBCL
Guo et al., 2019(22)	45/F	T1: isointense<br>T2: hypointense<br>DWI: no diffusion restriction	Bilateral ventricles and fornix	Small lymphocytic lymphoma
Guo et al., 2019(22)	49/M	T1: isointense<br>T2, Flair: hyperintense	Bilateral ventricles, thalamus, and callosal splenium	Small lymphocytic lymphoma
Wang at al. 2020(23)	51/F	Not available	The lateral, third and fourth ventricles	DLBCL
Ball et al., 2020(24)	75/F	T1, T2: hypointense<br>DWI: not available	The bilateral lateral ventricles	DLBCL
Ball et al., 2020(24)	60/M	T1, T2: hypointense<br>DWI: diffusion restriction	The left lateral, third and fourth ventricles	DLBCL
Ball et al., 2020(24)	74/M	T1, T2: hypointense<br>DWI: diffusion restriction	The bilateral lateral and third ventricles	DLBCL
Ball et al., 2020(24)	40/M	T1, T2: hypointense<br>DWI: diffusion restriction	The third ventricle and aqueduct	DLBCL
Ball et al., 2020(24)	64/F	T1, T2: hypointense<br>DWI: hyperintense and diffusion restriction	The third, fourth and left lateral ventricles	DLBCL
Kumar et al., 2020(25)	36/M	T1-enhanced: enhancement	The lateral, third, and fourth ventricles	DLBCL
Current case	78/M	T1: hypointense<br>T2: iso-hypointense<br>DWI: hypointense(the third ventricular lesion), diffusion restriction	The third and left lateral ventricles	DLBCL

References

1. Haegelen C, Riffaud L, Bernard M, Morandi X. Primary Isolated Lymphoma of the Fourth Ventricle: Case Report. *J Neurooncol* (2001) 51(2):129-31. Epub 2001/06/02. doi: 10.1023/a:1010790325692.
2. Hill CS, Khan AF, Bloom S, McCartney S, Choi D. A Rare Case of Vomiting: Fourth Ventricular B-Cell Lymphoma. *J Neurooncol* (2009) 93(2):261-2. Epub 2008/12/19. doi: 10.1007/s11060-008-9765-4.
3. Gu Y, Hou YY, Zhang XB, Hu F. Primary Central Nervous System Burkitt Lymphoma as Concomitant Lesions in the Third and the Left Ventricles: A Case Study and Literature Review. *J Neurooncol* (2010) 99(2):277-81. Epub 2010/02/11. doi: 10.1007/s11060-010-0122-z.
4. Takasu M, Takeshita S, Tanitame N, Tamura A, Mori M, Fujihara M, et al. Case Report. Primary Hypothalamic Third Ventriclular Burkitt's

Lymphoma: A Case Report with Emphasis on Differential Diagnosis. *Br J Radiol* (2010) 83(986):e43-7. Epub 2010/02/09. doi: 10.1259/bjr/84426981.
5. Sasani M, Bayhan M, Sasani H, Kaner T, Oktenoglu T, Cakiroglu G, et al. Primary Central Nervous System Lymphoma Presenting as a Pure Third Ventricular Lesion: A Case Report. *J Med Case Rep* (2011) 5:213. Epub 2011/05/31. doi: 10.1186/1752-1947-5-213.
6. Brar R, Prasad A, Sharma T, Vermani N. Multifocal Lateral and Fourth Ventricular B-Cell Primary Cns Lymphoma. *Clin Neurol Neurosurg* (2012) 114(3):281-3. Epub 2011/11/22. doi: 10.1016/j.clineuro.2011.10.020.
7. Bokhari R, Ghanem A, Alahwal M, Baeesa S. Primary Isolated Lymphoma of the Fourth Ventricle in an Immunocompetent Patient. *Case Rep Oncol Med* (2013) 2013:614658. Epub 2013/04/23. doi: 10.1155/2013/614658.
8. Rao RN, Mishra D, Agrawal P, Kumar R. Primary B-Cell Central Nervous System Lymphoma Involving Fourth Ventricle: A Rare Case Report with Review of Literature. *Neurol India* (2013) 61(4):450-3. Epub 2013/09/06. doi: 10.4103/0028-3886.117608.
9. Liao CH, Lin SC, Hung SC, Hsu SP, Ho DM, Shih YH. Primary Large B-Cell Lymphoma of the Fourth Ventricle. *J Clin Neurosci* (2014) 21(1):180-3. Epub 2013/09/10. doi: 10.1016/j.jocn.2013.02.036.
10. Funaro K, Bailey KC, Aguila S, Agosti SJ, Vaillancourt C. A Case of Intraventricular Primary Central Nervous System Lymphoma. *J Radiol Case Rep* (2014) 8(3):1-7. Epub 2014/06/27. doi: 10.3941/jrcr.v8i3.1361.
11. Fabiano AJ, Syriac S, Fenstermaker RA, Qiu J. Primary Fourth Ventricular B-Cell Lymphoma in an Immunocompetent Patient. *Clin Neuropathol* (2014) 33(1):94-7. Epub 2013/08/09. doi: 10.5414/NP300658.
12. Alabdulsalam A, Zaidi SZ, Tailor I, Orz Y, Al-Dandan S. Primary Burkitt Lymphoma of the Fourth Ventricle in an Immunocompetent Young Patient. *Case Rep Pathol* (2014) 2014:630954. Epub 2014/09/26. doi: 10.1155/2014/630954.
13. Grossman R, Nossek E, Shimony N, Raz M, Ram Z. Intraoperative 5-Aminolevulinic Acid-Induced Fluorescence in Primary Central Nervous System Lymphoma. *J Neurosurg* (2014) 120(1):67-9. Epub 2013/10/22. doi: 10.3171/2013.9.JNS131076.
14. Zhu Y, Ye K, Zhan R, Tong Y. Multifocal Lateral and Fourth Ventricular Primary Central Nervous System Lymphoma: Case Report and Literature Review. *Turk Neurosurg* (2015) 25(3):493-5. Epub 2015/06/04. doi: 10.5137/1019-5149.JTN.10496-14.1.
15. Hsu HI, Lai PH, Tseng HH, Hsu SS. Primary Solitary Lymphoma of the Fourth Ventricle. *Int J Surg Case Rep* (2015) 14:23-5. Epub 2015/07/26. doi: 10.1016/j.ijscr.2015.07.006.
16. Cellina M, Fetoni V, Baron P, Orsi M, Oliva G. Unusual Primary Central Nervous System Lymphoma Location Involving the Fourth Ventricle and Hypothalamus. *Neuroradiol J* (2015) 28(2):120-5. Epub 2015/04/30. doi: 10.1177/1971400915576671.
17. Suri V, Mittapalli V, Kulshrestha M, Premlani K, Sogani SK, Suri K. Primary Intraventricular Central Nervous System Lymphoma in an Immunocompetent Patient. *J Pediatr Neurosci* (2015) 10(4):393-5. Epub 2016/03/11. doi: 10.4103/1817-1745.174433.
18. Liu H, Hou H, Cheng J. Primary Burkitt Lymphoma of the Fourth Ventricle Mimicking a Medulloblastoma in a Child. *J Neurooncol* (2016) 127(1):205-7. Epub 2015/12/26. doi: 10.1007/s11060-015-2023-7.
19. Haddad R, Alkubaisi A, Al Bozom I, Haider A, Belkhair S. Solitary Primary Central Nervous System Lymphoma Mimicking Third Ventricular Colloid Cyst-Case Report and Review of Literature. *World Neurosurg* (2019) 123:286-94. Epub 2018/12/24. doi: 10.1016/j.wneu.2018.12.026.
20. Philippart M, Mulquin N, Gustin T, Fervaille C, London F. Primary Central Nervous System Lymphoma Revealed by Multiple Intraventricular Mass Lesions. *Acta Neurol Belg* (2019) 119(1):119-21. Epub 2018/09/05. doi: 10.1007/s13760-018-1017-6.
21. Brozovich A, Ewing D, Burns E, Hatcher C, Acosta G, Khan U, et al. Primary Cns Lymphoma Arising from the 4(Th) Ventricle: A Case Report and Review of the Literature. *Case Rep Oncol Med* (2019) 2019:2671794. Epub 2019/05/17. doi: 10.1155/2019/2671794.
22. Guo R, Zhang X, Niu C, Xi Y, Yin H, Lin H, et al. Primary Central Nervous System Small Lymphocytic Lymphoma in the Bilateral Ventricles: Two Case Reports. *BMC Neurol* (2019) 19(1):200. Epub 2019/08/21. doi: 10.1186/s12883-019-1430-3.
23. Wang D, Su M, Xiao J. A Rare Case of Primary Ventricular Lymphoma Presented on Fdg Pet/Ct. *Clin Nucl Med* (2020) 45(2):156-8. Epub 2019/12/14. doi: 10.1097/RLU.0000000000002876.
24. Ball MK, Morris JM, Wood AJ, Meyer FB, Kaszuba MC, Raghunathan A. Ventricle-Predominant Primary Cns Lymphomas: Clinical, Radiological and Pathological Evaluation of Five Cases and Review of the Literature. *Brain Tumor Pathol* (2020) 37(1):22-30. Epub 2019/10/21. doi: 10.1007/s10014-019-00354-x.
25. Kumar H, Sharma A, Sharma V, Singhvi S. Primary Central Nervous System Lymphoma Involving Entire Ventricular System. *Asian J Neurosurg* (2020) 15(1):126-7. Epub 2020/03/18. doi: 10.4103/ajns.AJNS_94_16.
